# Berberine induces autophagy in glioblastoma by targeting the AMPK/mTOR/ULK1-pathway

**DOI:** 10.18632/oncotarget.11396

**Published:** 2016-08-19

**Authors:** Jiwei Wang, Qichao Qi, Zichao Feng, Xin Zhang, Bin Huang, Anjing Chen, Lars Prestegarden, Xingang Li, Jian Wang

**Affiliations:** ^1^ Department of Neurosurgery, Qilu Hospital of Shandong University and Brain Science Research Institute, Shandong University, Jinan, 250012, P.R. China; ^2^ Department of Biomedicine, University of Bergen, 5009 Bergen, Norway; ^3^ Department of Dermatology, University of Bergen, 5021 Bergen, Norway

**Keywords:** berberine, glioblastoma, autophagy, apoptosis, metabolism

## Abstract

There is an urgent need for new therapeutic strategies for patients with glioblastoma multiforme (GBM). Previous studies have shown that berberine (BBR), a natural plant alkaloid, has potent anti-tumor activity. However, the mechanisms leading to cancer cell death have not been clearly elucidated. In this study, we show that BBR has profound effects on the metabolic state of GBM cells, leading to high autophagy flux and impaired glycolytic capacity. Functionally, these alterations reduce the invasive properties, proliferative potential and induce apoptotic cell death. The molecular alterations preceding these changes are characterized by inhibition of the AMPK/mTOR/ULK1 pathway. Finally, we demonstrate that BBR significantly reduces tumor growth *in vivo*, demonstrating the potential clinical benefits for autophagy modulating plant alkaloids in cancer therapy.

## INTRODUCTION

Glioblastoma multiforme (GBM) is the most aggressive primary brain tumor characterized by a highly infiltrative growth pattern and resistance to chemotherapy [1]. Despite multimodal treatment with surgery followed by radio- and chemotherapy with Temozolomide (TMZ), the 5-year survival rate of WHO grade IV glioblastoma is still less than 5% [2, 3]. As such, there is a critical need to identify new efficacious therapeutic strategies.

Berberine (BBR), an isoquinoline alkaloid isolated from Berberis vulgaris L., has been used extensively in traditional Chinese medicine to treat diarrhea and diabetes. Recent studies have shown that BBR also exerts anticancer activity towards a variety of cancer cell types, such as glioma, colorectal-, lung-, prostate- and ovarian cancer [4–10]. The cancer specific cytotoxic activity of BBR is mainly attributed to induction of apoptotic cell death characterized by Cytochrome C release followed by caspase-3 and -9 activation [11–14]. However, the mechanisms that underlie the induction of apoptosis by BBR are poorly delineated. [15, 16].

Autophagy maintains cellular homeostasis, and removes dysfunctional or damaged organelles that are digested and recycled for cellular metabolic needs [17]. Consequently, autophagy may support cancer survival under metabolic stress and mediate resistance to anticancer therapies such as radiation, chemotherapy and some targeted therapies [18]. Increasing evidence supports that inhibition of autophagy holds a therapeutic potential [19, 20]. Treatment with inhibitors of autophagy such as Bafilomycin A1 (Baf) and chloroquine (CQ) has been shown to potentiate the effects of several therapeutic agents [21, 22]. These studies have led to the initiation of multiple clinical trials combining chemotherapeutic agents and autophagy inhibitors for various cancer types [23]. However, recent studies have also demonstrated a therapeutic potential for enhancers of autophagy in GBM [24–26]. As such, the specific role of autophagy seems to be highly context- and cell type dependent. In this report, we explored the mechanisms leading to BBR induced cell death in GBM. We show that BBR induces autophagy and impairs the glycolytic capacity. Importantly, these changes reduce the invasive potential of GBM cells and induce cell death.

## RESULTS

### BBR inhibits cell proliferation in GBM cells

**Figure 1:**
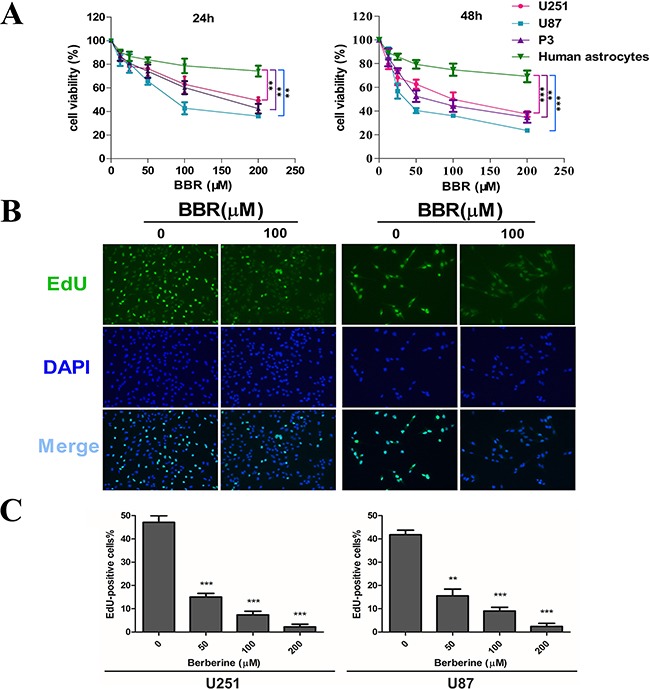
BBR inhibits cell proliferation in GBM cells **A.** A CCK-8 assay was performed to evaluate cell viability in P3, U251, U87 cells and human astrocytes treated with different concentrations of BBR for 24 and 48 h. **B.** EdU proliferation of U251 and U87 cells treated with different concentrations of BBR for 24 h. The cells were stained with Apollo 488 (green, representative of EdU) and nuclear specific dye DAPI (blue). **C.** Cell number and EdU content of U251 and U87 cells treated with different concentrations of BBR for 24 h. All data are expressed as the means ± SD of values from triplicate experiments. **p < 0.01 and ***p < 0.001 compared with control group.

To address the effect of BBR on glioma cell growth, we first evaluated cell viability using a CCK-8 assay (Figure 1A). Treatment with increasing concentrations of BBR resulted in growth inhibition of U251, U87 and P3 cells in a dose-dependent manner. Moreover, there was a significantly lower effect of BBR on normal human astrocytes. We next used the EdU incorporation assay to evaluate the BBRs effect on proliferation. As shown in Figure 1B-1C, BBR attenuated cell proliferation in both U251 and U87 cells in a dose-dependent manner.

### BBR induces apoptosis in GBM cells

**Figure 2:**
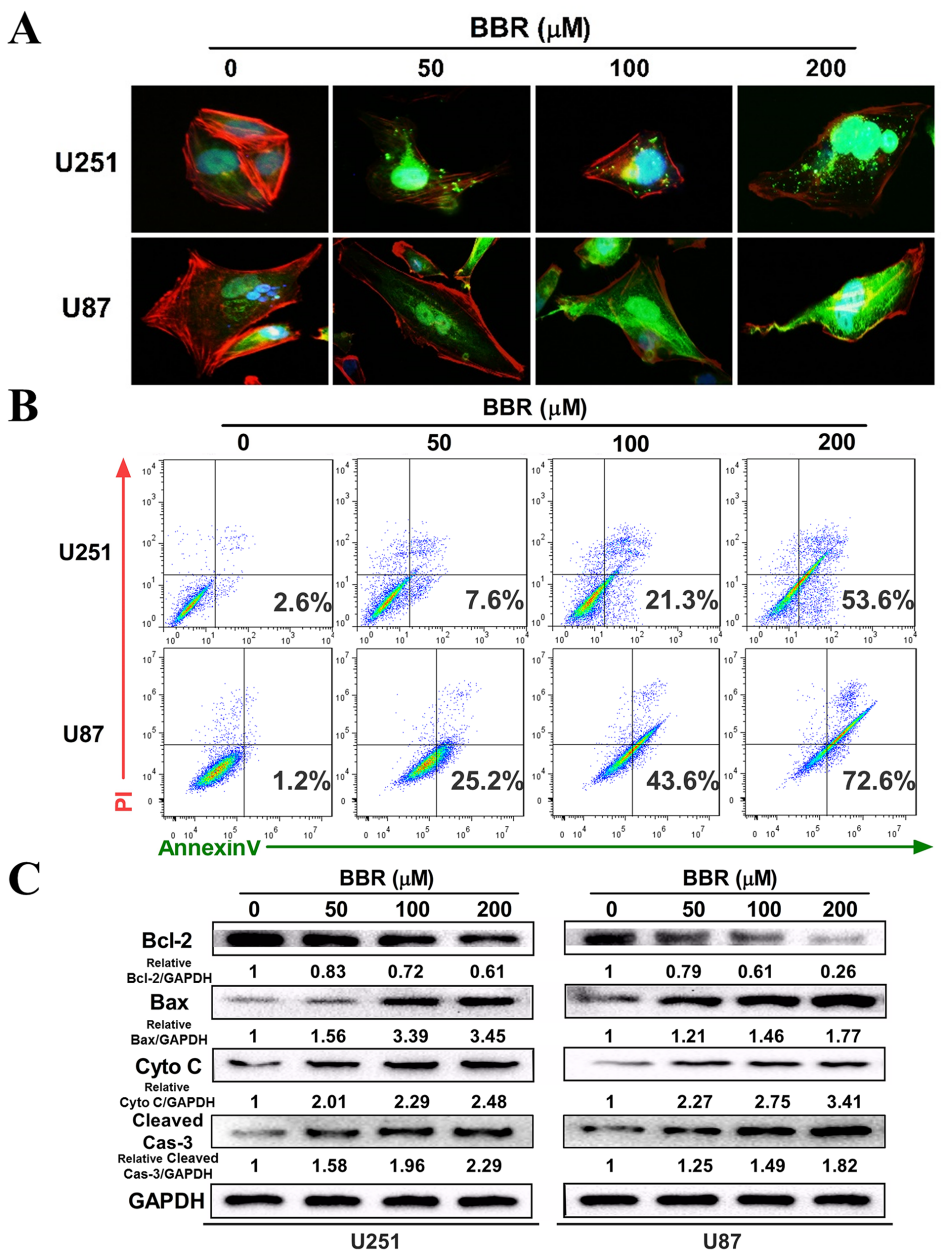
BBR induces apoptosis in GBM cells **A.** The expression of cytochrome C in the cytosol was detected using immunofluorescent imaging (IFI) analysis in U251 and U87 cells treated with different concentrations of BBR for 24 h. **B.** Annexin V/ PI staining of U251 and U87 cells treated with different concentrations of BBR for 24 h. **C.** Western blot analyses were performed to detect the protein expression of Bax, Bcl-2, cytochrome C, cleaved caspase-3 and GAPDH in U251 and U87 cells after treating with different concentrations of BBR for 24 h.

To investigate how BBR influences cancer cell viability we initially measured the expression of Cytochrome C by immunocytochemistry (ICC). There was a clear dose-dependent increase in Cytochrome C positive dots in BBR-treated cells compared to untreated (Figure 2A and Supplementary Figure S1). Next, we stained U87 and U251 with Annexin V/PI and quantified the number of cells undergoing apoptosis by flow cytometry. We found that BBR significantly induced apoptosis in both early (Annexin V + /PI −) and late (Annexin V + /PI +) stages (Figure 2B). To substantiate these findings we measured the expression of the mitochondrial apoptosis related proteins Bax, Cytochrome C and cleaved caspase-3, as well as the anti-apoptotic protein Bcl-2, in U251 and U87 cells by western blot analysis. Treatment with BBR up-regulated Bax, Cytochrome C, and cleaved caspase-3 and reduced the expression of Bcl-2 proteins compared with untreated U251 and U87 cells (Figure 2C). Taken together, these results supports that BBR reduces cell viability by inducing Cytochrome C mediated apoptotic cell death.

### BBR impairs migration and invasion in GBM cells

**Figure 3:**
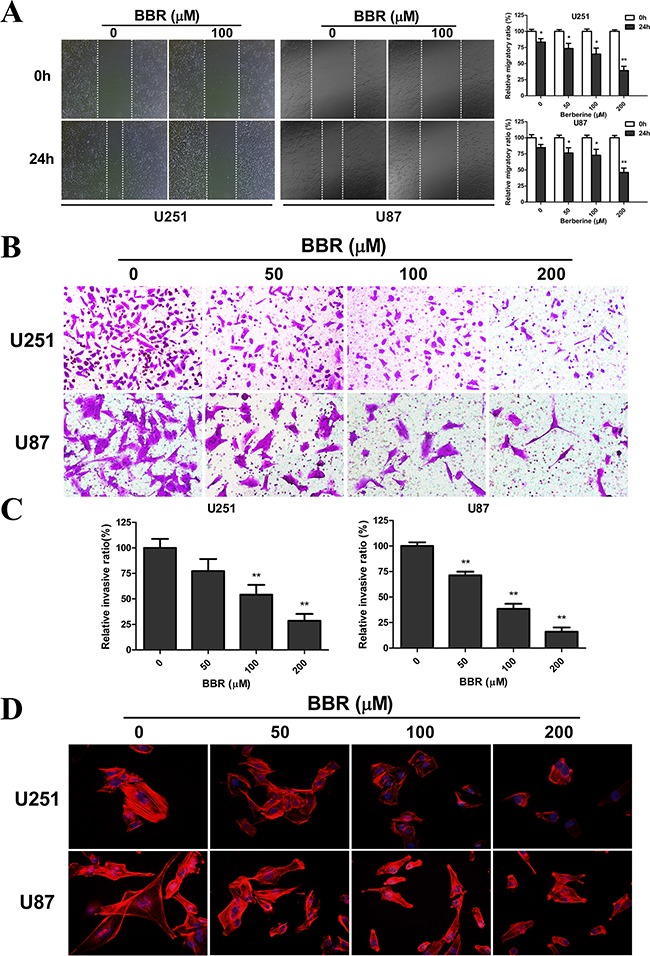
BBR impairs migration and invasion in GBM cells **A.** The migratory ability and statistical results of the migratory rate of U251 and U87 cells were evaluated by a wound healing assay treated with different concentrations of BBR for 24 h. **B.** Transwell results of U251 and U87 cells treated with different concentrations of BBR for 24 h. **C.** Statistical results of the invasive ratio treated with different concentrations of BBR for 24 h in the Transwell assay. **D.** Actin rearrangements were visualized using rhodamine phalloidin in U251 and U87 cells treated with different concentrations of BBR for 24 h. BBR-treatment resulted in actin impolarization. Actin ruffing at the edges of the elongated cells was also observed in untreated cells, indicating increased migration and invasion. All data are expressed as the mean ± SD of values from triplicate experiments. *p < 0.05, **p < 0.01 and ***p < 0.001 compared with control group.

Having observed a profound change in cell morphology during treatment with BBR, with retraction of pseudopodia, we used the wound healing assay to examine whether BBR affected migration. Untreated GBM cells had a significant higher migratory rate than the BBR-treated cells both in U251 and U87 (Figure 3A). The Transwell assay was used to evaluate the inhibitory effect of treatment with BBR on cell invasion. U251 and U87 GBM cells were plated in upper chambers coated with Matrigel, which mimics the extracellular matrix (ECM) around tumors. As shown in Figure 3B-3C, cancer cell invasion was significantly reduced through the transwell chamber micropores in the BBR treatment group (Figure 3B and 3C). An important characteristic of advanced cancer is the rearrangement of the cytoskeleton of migrating and invading cells [27]. To address this in our model system we used rhodamine phalloidin staining. Untreated GBM cells were characterized by more organized F-actin filaments compared to BBR-treated cells (Figure 3D). Thus, these results supports that BBR impairs the migratory and invasive potential in GBM cells.

### BBR reduces the glycolytic capacity in GBM cells

**Figure 4:**
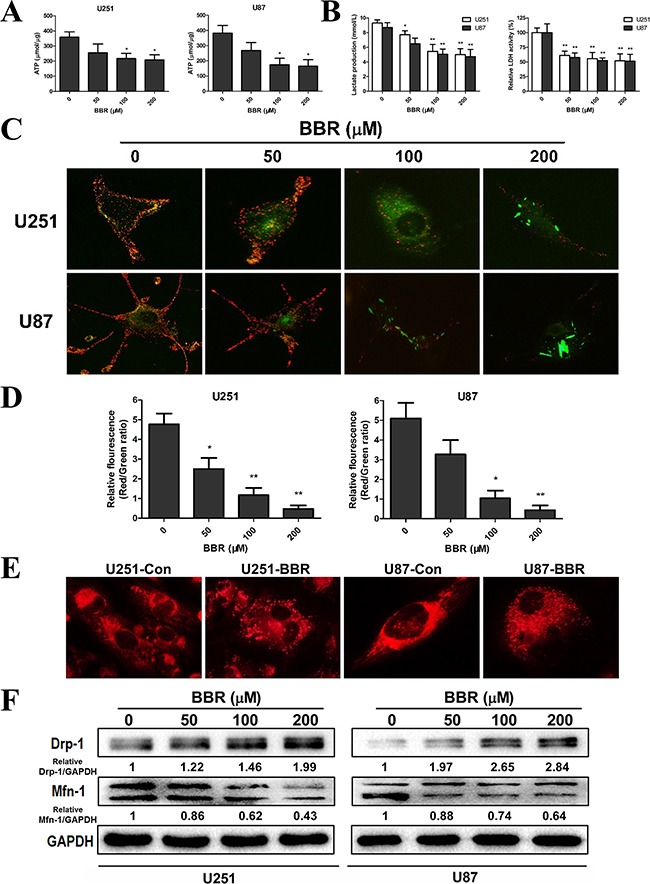
BBR reduces the glycolytic capacity in GBM cells **A.** ATP synthesis level in U251 and U87 cells treated with different concentrations of BBR for 24 h. **B.** L-lactate production and the corresponding LDH activity of U251 and U87 cells treated with different concentrations of BBR for 24 h. **C.** Changes in mitochondrial membrane potential (△Ψm) in U251 and U87 cells using the JC-1 probe treated with different concentrations of BBR for 24 h. **D.** Quantitative analysis of △Ψm was conducted by ratio of Red: Green fluorescence (ratio of 590:530 nm emission intensity). **E.** Changes inmitochondrial morphology were visualized with MitoTracker Red in U251 and U87 cells treated with or without BBR (100 μM) for 24 h, focusing on the conversion between fusion and fission. **F.** Western blot analyses were performed to detect the protein expression changes of Mfn1, Drp 1 and GAPDH in U251 and U87 cells treated with different concentrations of BBR for 24 h. All data are expressed as the means ± SD of values from triplicate experiments. *p < 0.05, **p < 0.01 and ***p < 0.001 compared with control group.

Tumor cell invasion is a highly energy consuming process [28, 29]. A shift from mitochondrial respiration to glycolysis, the Warburg effect, is one of the most prominent metabolic alterations in cancer cells and has been show to enhance cancer cell invasion [30]. To assess the impact of BBR on the metabolic hemostasis we measured the ATP levels in GBM cell lines. The ATP level was significantly reduced in U251 and U87 cells after BBR treatment (Figure 4A). We then measured L-lactate, the main metabolic product of glycolysis, in the cell culture media in glioma cells. As shown in Figure 4B, BBR attenuated L-lactate levels of cell culture media in both U251 and U87 cells in a dose-dependent manner. Corresponding results were found for LDH, further strengthening that BBR reduced the glycolytic activity (Figure 4B). A reduction in total ATP levels could indicate reduced compensatory mitochondrial capacity. To address whether BBR also affected the mitochondrial capacity we used the JC-1 assay. JC-1 is an indicator of mitochondrial membrane potential that forms J-aggregates with red fluorescence in high △Ψm conditions and J-monomers with green fluorescence in low△Ψm conditions during which the conversion between red and green directly reflects changes in △Ψm [31]. We observed green fluorescence increasing after BBR treatment in U251 and U87 cells (Figure 4C), and this was substantiated by the ratio of Red/Green fluorescence, which showed that the percentage of green was increased in BBR-treated cells, indicating decreased oxidative phosphorylation in BBR-treated U251 and U87 cells (Figure 4D). We next asked whether the functional alterations were reflected in morphological changes. Based on their size, mitochondria can be classified morphologically as fragmented, intermediate or tubular [32]. Based on data from MitoTracker staining The tubular form is a more favorable form in during cell [33]. We calculated the percentages of the three forms in U251 and U87 cells and found that the BBR-treated cells favored more fragmented mitochondria, while untreated cells formed tubular mitochondria, indicating alterations in the fusion to fission process in BBR-treated cells (Figure 4E). The mitochondrial protein Mfn1 is required for mitochondrial fusion, while Drp 1 plays a central role in mitochondrial fission [34]. Western blot for Mfn1 and Drp1 supported our findings of altered mitochondrial dynamics (Figure 4F). Taken together, these results demonstrate that BBR attenuates glycolysis dependent energy production and induced mitochondrial dysfunction.

### BBR induces autophagy in GBM cells

**Figure 5:**
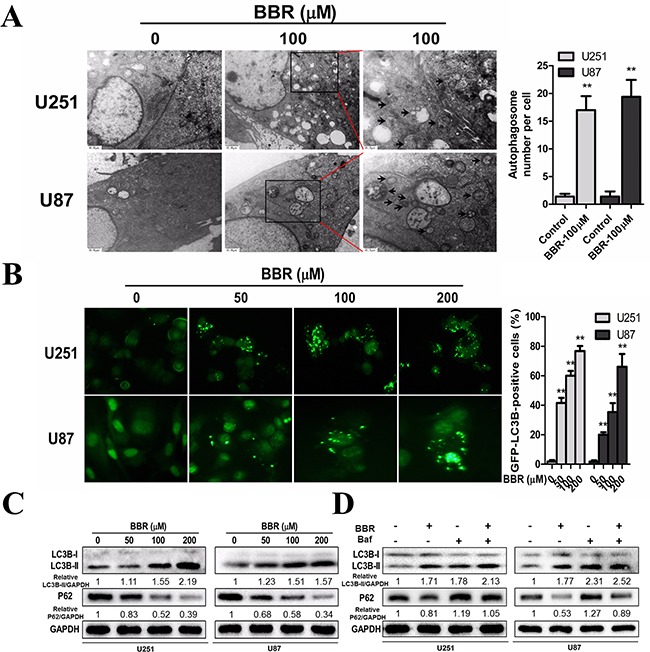
BBR induces autophagy in GBM cells **A.** Transmission electron microscopy showed the formation of autophagosomes in U251 and U87 cells after BBR treatment for 24 h. Representative autophagosomes are shown in the enlarged image (arrows). The number of autophagosomes per cell were quantified. **B.** U251 and U87 cells stably expressing GFP-LC3B from lentiviruses were treated with different concentrations of BBR for 24 h. The percentage of cells with more than four GFP-LC3B dots were quantified. **C.** Western blot analyses showed the dose-dependent effect of BBR treatment for 24 h on the endogenous conversion of LC3 and the amount of SQSTM1/p62 in U251 and U87 cells. The LC3B-II/GAPDH and p62/GAPDH ratios were calculated using ImageJ 1.36b. **D.** LC3B and p62 levels were examined by Western blot analysis for U251 and U87 cells after treatment with BBR (100 μM) or DMSO in the absence or presence of Baf (100 nM) for 24 h. The LC3B-II/GAPDH and p62/GAPDH ratios were calculated using ImageJ 1.36b. All data are expressed as the means ± SD of values from triplicate experiments. **p < 0.01 compared with control group.

Previous studies have suggested that BBR exerts its anticancer effect by inducing autophagy [11–14]. We therefore investigated the exact relationship between BBR and autophagy in the U251 and U87 GBM cell lines. Transmission electron microscopy (TEM) is the gold standard for identifying autophagosome formation, which is characterized by their double-membrane structure. TEM analysis clearly demonstrated increased production of autophagosomes after BBR treatment (Figure 5A). To further address the role of BBR-induced autophagy in GBM cells, we generated stably GFP-LC3 expressing GBM cells. The percentage of GFP-LC3 positive puncta increased in BBR-treated cells in a dose-dependent manner (Figure 5B). To examine whether BBR treatment induced or impaired autophagy, we measured the levels of LC3B-II and SQSTM1/p62 following BBR treatment. LC3B-II, a marker for autophagy, increased in a dose-dependent manner in GBM cells (Figure 5C). Consistent with this, the protein levels of SQSTM1/p62 decreased, indicating enhanced autophagy flux. Consistent with these observations, incubation with Bafilomycin A1 (Baf), which inhibits late stage autophagy, increased the levels of both SQSTM1/p62 and LC3B-II, while a combination of BBR and Baf enhanced the LC3-II level and resulted in intermediate levels of SQSTM1/p62 (Figure 5D). Overall, these data indicate that BBR induces autophagy in GBM cells.

### BBR targets the AMPK/mTOR/ULK1 pathway in GBM cells

**Figure 6:**
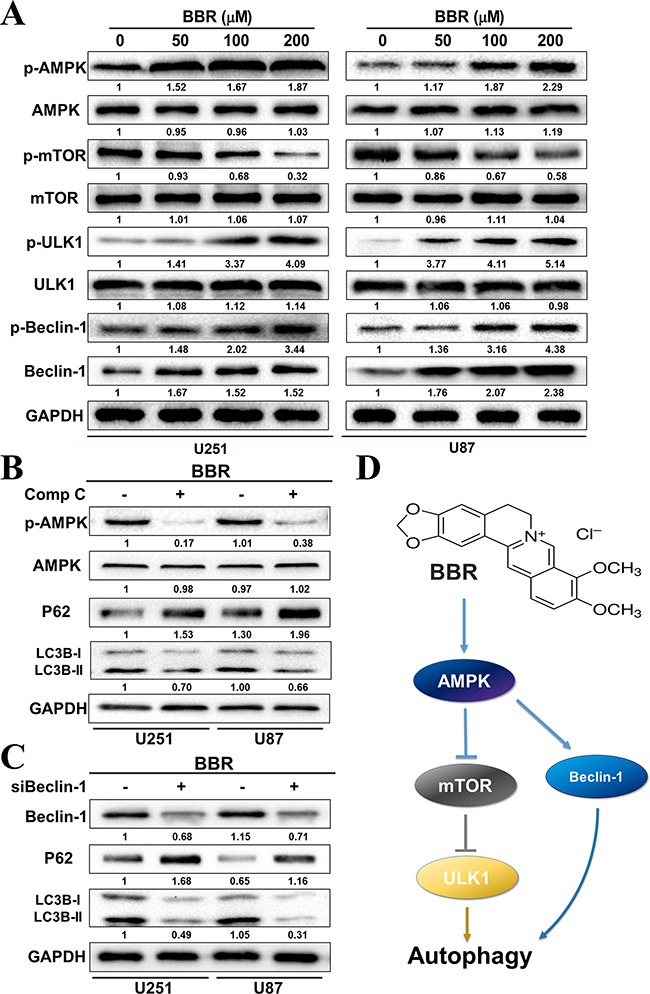
BBR targets the AMPK/mTOR/ULK1 pathway in GBM cells **A.** Western blot analysis of p-AMPK (Thr172), AMPK, p-mTOR (Ser2448), mTOR, p-ULK1 (Ser555), ULK1, p-Becin-1 (Ser93) and Beclin-1 expression of U251 and U87 cells treated with different concentrations of BBR for 24 h. **B.** Western blot analysis of p-AMPK (Thr172), AMPK, p62, and LC3B treated with Comp C (20 μM) or DMSO in U251 and U87 cells after treatment with BBR (100 μM) for 24h. **C.** Western blot analysis of Beclin-1, p62, and LC3Bin Beclin-1 knockdown or non-Beclin-1 knockdown U251 and U87 cellsafter treatment with BBR (100 μM) for 24h. **D.** Schematic diagram to illustrate the whole process by which BBR triggered autophagy in glioma cells.

The mammalian target of Rapamycin (mTOR) is a key regulator of autophagy. Previous studies has shown that activation of mTOR impairs autophagy and pharmacological mTOR inhibitors induce autophagy in most model systems [35]. In our experiments, we observed that BBR dephosphorylated mTOR in a dose-dependent manner in both U251 and U87 cells (Figure 6A), indicating that mTOR suppression contributed to the induction of autophagy by BBR. The AMP-activated protein kinase (AMPK) is activated by alterations in the cellular energy hemostasis and previous studies have shown that BBR can activate AMPK [36]. Activated AMPK negatively regulates mTOR and thereby enhances autophagy flux. As shown in Figure 6A, there is a dose dependent increase in AMPK protein levels upon BBR treatment. This was followed by increased levels of Beclin-1, which is directly dependent on phosphorylation at Ser93 or Ser96 by AMPK. We then found that BBR-treatment increased the level of p-Beclin-1 Ser93 in GBM cells. To address whether BBR activation of autophagy was AMPK dependent we incubated GBM cells with the AMPK inhibitor Compound C. This reduced the levels of p-AMPK and attenuated BBR-induced autophagy flux (Figure 6B). We then examined the effects of knockdown of Beclin-1 on BBR-induced autophagy. As shown in Figure 6C, knockdown of Beclin-1 prevented BBR-induced autophagy flux compared with the siRNA control group. Finally, BBR induced a dose-dependent increase in the levels of phosphorylated ULK1, which is a downstream target of mTOR. These findings indicated that AMPK/mTOR/ULK1 pathway is involved in BBR-induced autophagy in GBM cells.

### BBR reduces tumor growth *in vivo*

**Figure 7:**
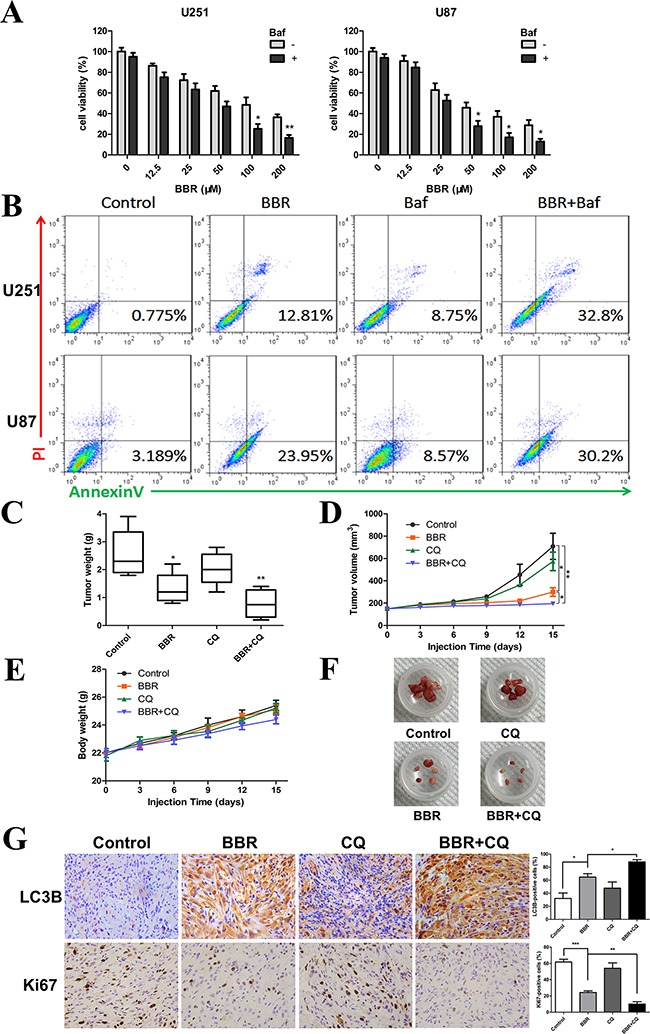
BBR reduces tumor growth *in vivo* **A.** A CCK-8 assay was performed to assess cell viability in U251 and U87 cells treated with different concentrations of BBR in the presence or absence of Baf (100 nM) for 24 h. **B.** Annexin V/ PI staining of U251 and U87 cells treated with BBR (100 μM) or DMSO in the absence or presence of Baf (100 nM) for 24 h. **C-F.** Mice were sacrificed 18 days after the indicated treatments, and tumor weight, tumor volume, and mouse body weight were measured. **G.** Immunohistochemical staining for LC3B and Ki67 of tumors from each group. The charts indicate the percentage of LC3B or Ki-67 positive cells. All data are expressed as the means ± SD of values from triplicate experiments. *p < 0.05, **p < 0.01 and ***p < 0.001 compared with control group.

Some studies have suggested that autophagy serves a protective role in tumor cells and that therapy-induced cell death could be enhanced by autophagy inhibition [37]. To determine the role of autophagy in BBR-treated glioma cells, the autophagy inhibitor Baf (for *in vitro*) and CQ (for *in vivo*) were used to prevent autophagy at a late stage. As shown in Figure 7A and 7B, Baf significantly enhanced the BBR-induced apoptosis of glioma cells *in vitro*. To determine the potential therapeutic efficiency of BBR *in vivo*, athymic nude mice were implanted with U87 cells and assigned to the following treatment groups: Control, BBR, CQ and BBR combined with CQ. Our results showed that BBR monotherapy significantly reduced tumor growth (Figures 7C, 7D, and 7E). Although no statistically significant differences were found between the CQ and control arms, BBR combined with CQ treatment was more effective compared BBR alone (Figures 7C, 7D and 7E). There was no significant body weight loss in the BBR or combination treatment groups compared to the control group (Figure 7E). Western blot analysis of tumor tissue confirmed that p-AMPK was upregulated and p-mTOR downregulated during BBR treatment *in vivo* (Supplementary Figure S2). Immunohistochemistry (IHC) analyses of tissue sections from the combination group showed that the LC3B levels were increased while proliferation, as determined by Ki-67 positive cells, was decreased (Figure 7G). H&E staining of the livers from all four groups (Supplementary Figure S3) did not reveal any difference in histology, suggesting that neither BBR monotherapy nor combination treatment was toxic to the mice.

## DISCUSSION

Previous studies have provided compelling evidence for the anti-cancer effects of BBR in different cancer types [4–10]. However, the mechanistic basis for this effect is poorly understood in GBM cells. In the present study we show that BBR suppresses GBM cell metabolism, leading to reduced glycolytic capacity, altered mitochondrial dynamics and increased autophagy flux. Autophagy maintains cancer survival during metabolic stress, and numerous anti-cancer agents have been reported to modulate cellular autophagy [38–40]. However, while some studies have shown that autophagy-inducing compounds have anti-proliferative effects [25], others have shown that high autophagy flux is beneficial for cancer cells. In the present study, we showed that the cytotoxic effects of BBR is characterized by accumulation of LC3B-II positive autophagosomes followed by reduced p62 protein levels, indicating increased autophagy in our model system.

Autophagy is regulated by a complex signaling network, most of which feed into the AMPK/mTOR pathway, The serine/threonine kinase mTOR acts as a negative gatekeeper of autophagy, and compounds that trigger autophagy can be broadly divided into two groups: mTOR-dependent and mTOR-independent. Our data showed that BBR suppressed the level of p-mTOR and increased AMPK levels in both U251 and U87 cells, indicating that BBR induced mTOR-dependent autophagy in GBM cells.

The proapoptotic effects of autophagy are highly context dependent and the interplay between autophagic and apoptotic signaling pathways is complex. Some stress signals may induce both pathways simultaneously. However, in normal cells, autophagy usually inhibits apoptosis and vice versa. Drug induced supra-physiological autophagy may, on the other hand, induce apoptosis by excessive degradation of cytoplasmic organelles. In the present study, we observed a survival benefit in tumor bearing animals by enhancing autophagy flux with BBR and simultaneously blocking the fusion of autophagosomes with the lysosomes. Even though the specific mechanisms for this additive effect are unclear, recent reports supports that this combination might lead to toxic accumulation of autophagosomes [39, 40]. As such, this provides the rationale for inducing early stage autophagy while impairing the autophagosome-lysosome function.

In summary, our work shows that BBR targets GBM cells both *in vitro* and *in vivo*, and support further assessment of using BBR in the adjuvant clinical setting for GBM.

## MATERIALS AND METHODS

### Reagents

Berberine chloride (BBR), Bafilomycin A1 (Baf), chloroquine (CQ), AMPK inhibitor Compound C (Comp C) and Dimethyl sulfoxide (DMSO) were obtained from Sigma-Aldrich, USA (PHR1502, B1793, C6628, P5499, D2650). The structure of berberine has been demonstrated in a review [16]. The following primary antibodies were used: Rabbit anti-LC3B, SQSTM1 (p62), Cytochrome C, Bcl-2, Drp-1, AMPK, p-AMPK (Thr172), mTOR, p-mTOR (Ser2448), ULK1, p-ULK1 (Ser555), Beclin-1, p-Beclin-1 (Ser93), Ki67, and GAPDH antibodies were purchased from Cell Signaling Technology. Rabbit anti-mitofusion-1 and Bax were purchased from Abcam. Rabbit anti-cleaved-caspase-3 (p17) was purchased from Santa Cruz Biotechnology.

### Cell lines and cultures

Human glioma cell lines (U251 and U87) were purchased from the Chinese Academy of Sciences Cell Bank (Shanghai, China). Human glioma P3 cell line and human astrocytes were kindly provided by Prof. Rolf Bjerkvig, University of Bergen. Both U251, U87, P3 and human astrocytes cells have been recently authenticated based on cross species checks, DNA authentication and quarantine. The cell lines (U251 and U87) were grown in Dulbecco's modified Eagle's medium (DMEM, SH30022.01B, Hyclone, UT, USA) supplemented with 10% fetal bovine serum (10082147, Gibco, MD, USA) in a humidified incubator with 5% CO_2_ at 37°C. Human astrocytes were incubated in an astrocyte medium consisting of 20% fetal bovine serum and 1% astrocyte growth supplement (ScienceCell) in humidified air with 5% CO_2_ at 37°C.

### Cell viability and proliferation assays

Cell viability was assessed with a Cell Counting Kit-8 (CCK-8; CK04-500, Dojindo, Kumamoto, Japan). Tumor cells in medium containing 10% fetal bovine serum were seeded into 96-well, flat-bottomed plates at 5×10^3^ cells/well and incubated at 37°C overnight. After the desired treatment, cells were incubated for an additional 4 h with 100 μl of serum-free DMEM with 10 μl of CCK-8 at 37°C. The absorbance at 450 nm was measured using a microplate reader (Bio-Rad, USA). Proliferation was examined using the EdU incorporation (C103103, Ribobio, Guangzhou, China) assay, which was performed according to the manufacturer's protocol, and the cells were examined under a fluorescence microscope.

### Cell migration and invasion assays

Cell migration was assessed with wound healing assays. U251 or U87 glioma cells were seeded into 6-well, flat-bottomed plates and incubated at 37°C overnight. A cell-free gap was generated by scratching with a 200 μl pipette tip. The wound closure area was monitored at different time points under a microscope and quantified using ImageJ software. Cell invasion was examined using Boyden chamber assays. The bottom of the Transwell membrane was pretreated with Matrigel (Becton-Dickinson, Bedford, MA, USA) for 4 h. Afterwards, 5×10^4^ cells re-suspended in serum-free DMEM were plated in the upper chamber of a trans-well apparatus (8.0 μm pore, Corning), while 600 μL of DMEM with 10% FBS were provided in the lower chamber. After incubation at 37°C for 24 h, cells that migrated to the bottom of the membrane were attached and fixed, stained with 0.5% crystal violet, and cells in the upper chamber were removed with a cotton sticker. The images were acquired using a light microscope. The cytoskeleton changes in U251 and U87 were visualized using rhodamine phalloidin (PHDR1, Cytoskeleton, Inc.) which was performed according to the manufacturer's protocol, and the cells were examined under a fluorescence microscope.

### Mitochondrial morphology and measurement of △Ψm

For the mitochondrial morphology, live cells were fluorescently labeled with 25 nM MitoTracker Red (Invitrogen, Molecular Probes). Mitochondrial morphology was then analyzed using an Olympus BX61 fluorescence microscope. Pictures were scanned using a DP71 CCD (charge-coupled device) digital camera. To determine △Ψm measurements, the cells were loaded with 50 nM JC-1 (5, 5, 6, 6-tetrachloro-1, 1, 3, 3-tetraethylbenzimidazolylcarbocyanineiodide, CaymanChemicals) for 30 min at room temperature according to the manufacturer's recommended protocol. The mitochondrial membrane potential was analyzed using an Olympus BX61 fluorescence microscope and fluorescence intensity ratio of JC-1 aggregates to JC-1 monomers (ratio of 590:530 nm emission intensity) [41]. Pictures were scanned using a DP71 CCD (charge-coupled device) digital camera.

### Western blotting

U251 and U87 glioma cells were harvested and washed with cold PBS. Total protein was extracted from cells using RIPA buffer (P0013B, Beyotime, Shanghai, China) with 1% phenylmethyl sulfonylfluoride, and protein concentrations were then determined by the BCA method (23225, Beyotime). Proteins were separated using 8–15% SDS-PAGE and transferred onto PVDF membranes (ISEQ00010 0.22 μm, Millipore, USA). For staining, each membrane was blocked for 1 h at room temperature (RT) with 5% skim milk in TBST (20 mmol/l Tris-HCL (pH 8.0), 137 mmol/l NaCl and 0.1% Tween-20 or with 5% BSA in TBST for phospho-proteins). Primary antibodies were incubated overnight at 4°C. After incubation, membranes were washed with TBST and re-probed with the appropriate horseradish peroxidase (HRP)-conjugated secondary antibodies, anti-mouse immunoglobulin G (IgG), anti-rabbit IgG or anti-goat IgG (1:5,000; Santa Cruz Biotechnology) for 1 h at RT. The proteins were visualized using Millipore's enhanced chemiluminescence (ECL) and detection system (ChemiDoc Touch, BioRad).

### Measurement of ATP, lactate and lactate dehydrogenase (LDH)

The total ATP, lactate and LDH levels were measured by plating 2 × 10^5^ cells in 6-well plates overnight, and then the cells were counted. Serum and the culture medium samples were collected for ATP, lactate and LDH assays. The total ATP levels were determined using the Cell Titer-Glo Luminescent assay (Promega) according to the manufacturer's instructions. Lactate was measured using the L-Lactate colorimetric assay kit (Abcam), and LDH was measured using a colorimetric LDH Assay kit (Abcam) following the manufacturer's recommended protocol. The data were normalized to the number of cells.

### Immunofluorescence

U251 or U87 cells were plated on glass slides in 24-well culture plates at a concentration of 2 × 10^5^ cells/well for 24 h and were subsequently treated with drugs for an additional 48 h in DMEM containing 10% FBS. The cells were then fixed with a 4% formaldehyde solution in PBS, permeabilized with 0.5% Triton X-100 in PBS, stained with rabbit anti-Cytochrome c antibody at a dilution of 1:400 in 5% bovine serum albumin in PBS overnight, and labeled with anti-rabbit IgG conjugated with FITC (Santa Cruz Biotechnology). The cells were incubated in the dark with phalloidin and DAPI. Slides were then examined under an Olympus BX61 fluorescence microscope. Pictures were scanned using a DP71 CCD (charge-coupled device) digital camera.

### Flow cytometric analysis of apoptosis

U251 and U87 glioma cells were harvested and re-suspended in a binding buffer. Cells were then stained with Annexin V-FITC (BD bioscience) according to the manufacturer's instructions. Cells were analyzed by a flow cytometry (Novocyte, ACEA), and the data were analyzed with Flowjo Software (Tree Star, Ashland, OR, USA).

### Constructs, transfection, and lentiviral infection

GFP-LC3 (pBABEpuro, 22405)-expressing vectors were obtained from Addgene (Cambridge, MA, USA). Lentiviral supernatants were prepared according to the manufacturer's instructions and provided by GenePharma. Lentiviral infections were carried out accordingly. To quantify autophagic cells after BBR treatment, we counted the number of autophagic cells demonstrated by GFP-LC3 dots (≥3 dots as a positive cell) [42]. Pictures were scanned with a DP71 CCD digital camera.

### Transmission electron microscopy

Cells were fixed with 4% glutaraldehyde and postfixed with 1% OsO4 in 0.1 M cacodylate buffer containing 0.1% CaCl_2_ for 2 h at 4°C. The samples were then stained with 1% Millipore-filtered uranyl acetate, dehydrated in increasing concentrations of ethanol, infiltrated, and embedded in LX-112 medium (Ladd Research Industries, Inc.). After polymerization of the resin at 60°C for 48 h, ultrathin sections were cut with an ultracut microtome (Leica). Sections were stained with 4% uranyl acetate and lead citrate, and images were obtained using a JEM-100cxII electron microscope (JEM).

### Small interfering RNA transfection

Beclin-1 siRNA (siBeclin-1) and negative control siRNA were designed and purchased by Gene Pharma (Shanghai, China). The siRNAs were transfected with Lipofectamine 2000 (11668-019, Life Technologies, CA, USA) in U251 and U87 cells according to the manufacturer's instructions. The sequences of siBeclin-1 were as follows: 5′-GGAGCCAUUUAUUGAAACUTT-3′ (sense) and 5′-GUUUCAAUAAAUGGCUCCTT-3′ (antisense).

### Animal studies

Athymic mice (male; 4 weeks old; 20–30 g), shaved of their outer fur, were provided by Shanghai SLAC Laboratory Animal Co., Ltd (Shanghai, China). The mice were randomly divided into four groups (control group, *n* = 5; BBR group, *n* = 5; CQ group, *n*=5; BBR+CQ group, *n*=5). U87 glioma cells (2×10^6^) in 100 μl of serum-free DMEM were inoculated subcutaneously into the right flanks of the mice.

Mice were gavaged with PBS alone (control), BBR (50 mg/kg/day), CQ (25 mg/kg/day) and BBR (50 mg/kg/day) plus CQ (25 mg/kg/day) every other day starting on day 3. Tumor volume was measured every third day with calipers and calculated as (L × W^2^) / 2, where L was the length and W was the width. Mice were euthanized after 18 days. Tumors were dissected and frozen in liquid nitrogen or fixed in formalin. All animal procedures were approved by the Institutional Animal Care and Use Committee (IACUC) of Shandong University.

### Immunohistochemistry

Solid tumors were removed from sacrificed mice and fixed with 4% formaldehyde. Paraffin-embedded samples were sliced and mounted on microscopic slides. Heat-induced epitope retrieval was performed with a microwave in 10 mmol/L citric acid buffer at pH 7.2. The samples were incubated at 4°C overnight with the primary antibody (rabbit anti-LC3B 1:200 dilutions; rabbit anti-Ki67 1:200 dilutions). Next, the sections were rinsed with PBS and incubated with horse-radish peroxidase-linked goat anti-rabbit, followed by reaction with diaminobenzidine and counter staining with Mayer's hematoxylin.

### Statistical analysis

All results are expressed as the mean ± standard deviation (S.D.). Data were obtained from three independent experiments. All statistical analyses were conducted using GraphPad Prism 5 software (San Diego, CA). Data were analyzed using paired t-tests. Significant differences: *p < 0.05; **p < 0.01;***p < 0.001.

## SUPPLEMENTARY MATERIAL FIGURES


