# Supplementary material for: Berberine induces autophagy in glioblastoma by targeting the AMPK/mTOR/ULK1-pathway
Source: Oncotarget. 2016 Aug 19;7(41):66944–58. doi: 10.18632/oncotarget.11396 (PMC5341849; doi:10.18632/oncotarget.11396)
Supplement: Supplementary file 1 [file oncotarget-07-66944-s001.pdf]

Berberine induces autophagy in glioblastoma by targeting the AMPK/mTOR/ULK1-pathway

SUPPLEMENTARY FIGURES

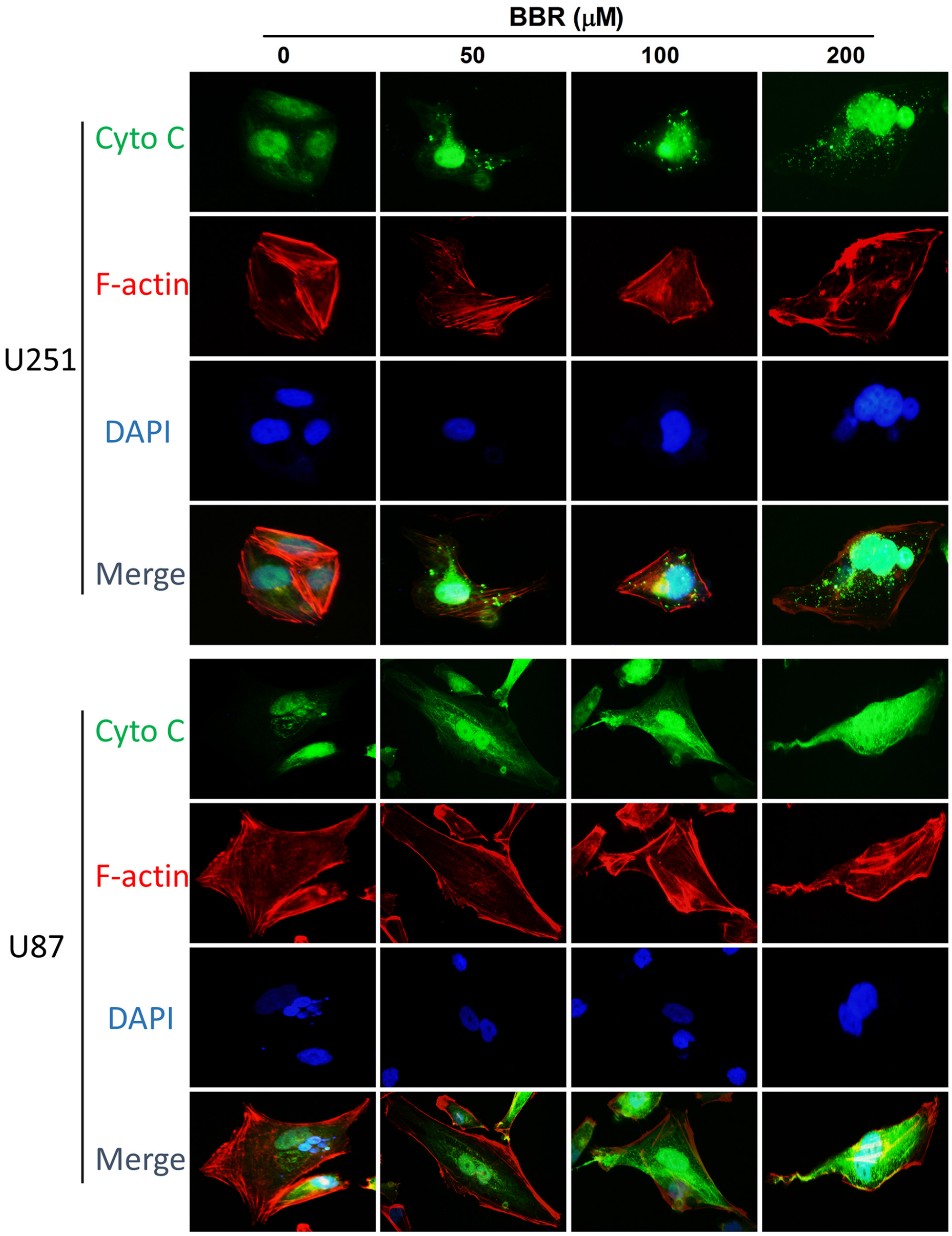

Supplementary Figure S1: BBR increases the expression of Cytochrome C.

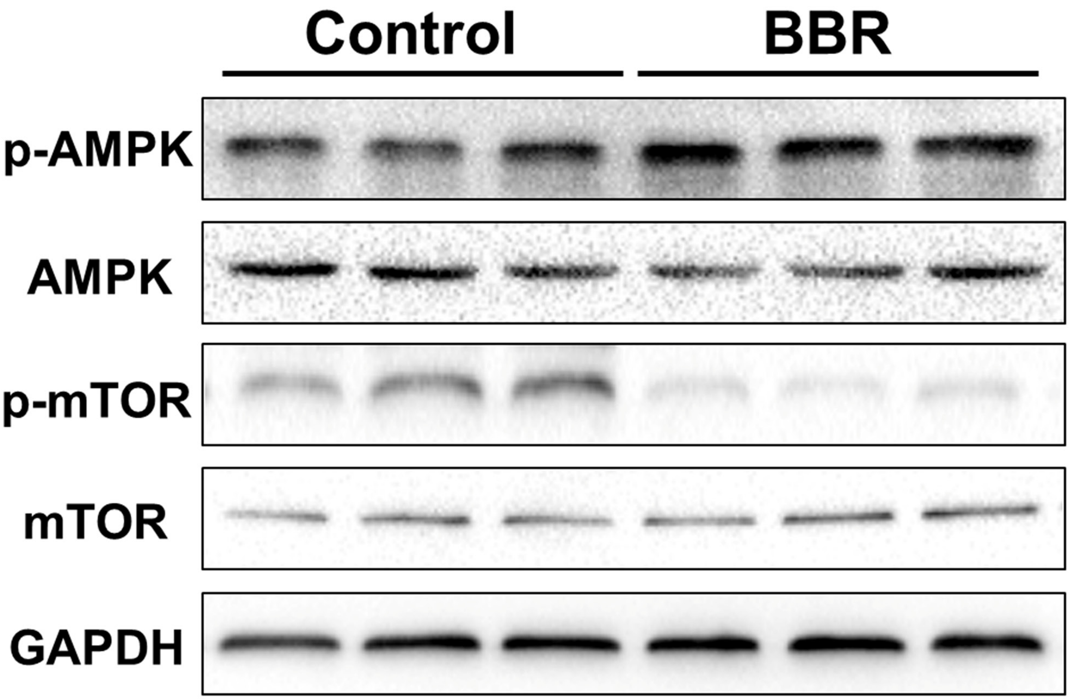

Supplementary Figure S2: Western blot of AMPK/mTOR signaling pathway in BBR-treated mice.

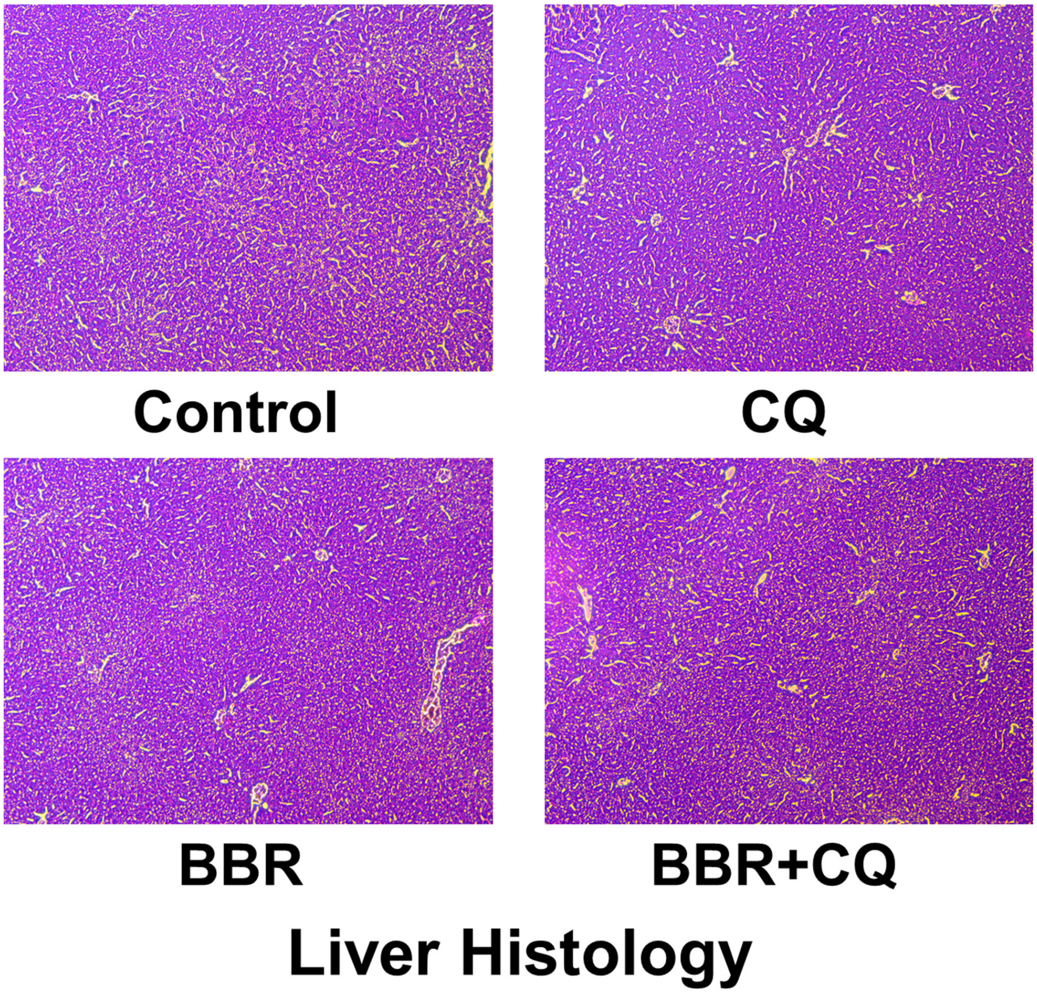

Supplementary Figure S3: Histology of H and E stained liver from BBR-treated and untreated mice. Gross histological examination reveals no apparent pathological alterations.
